# RNA•DNA:DNA triplex formation by *HIF1α-AS1* modulates individual base pair stabilities in the *adrenomedullin* DNA target duplex

**DOI:** 10.1261/rna.080909.125

**Published:** 2026-08

**Authors:** Nina M. Krause, Julia Wirmer-Bartoschek, Christian Richter, Matthias S. Leisegang, Ralf P. Brandes, Harald Schwalbe

**Affiliations:** 1 Center for Biomolecular Magnetic Resonance (BMRZ), Institute for Organic Chemistry and Chemical Biology, Johann Wolfgang Goethe University, Frankfurt am Main 60438, Germany; 2 Institute for Cardiovascular Physiology, Johann Wolfgang Goethe University, German Centre of Cardiovascular Research (DZHK), Partner site Rhine-Main, Frankfurt, Hesse 60596, Germany

**Keywords:** RNA•DNA:DNA triplex, NMR spectroscopy, base pair stabilities

## Abstract

Long noncoding RNAs (lncRNAs) play key roles in gene regulation. One potential regulation mechanism involves the formation of RNA•DNA:DNA triplexes. In these triplexes, the lncRNA binds in the major groove of a target DNA via Hoogsteen base pair formation. Here, we investigated the impact of the underlying RNA binding on the stability of the DNA duplex target to gain insights into the triplex stability at base pair resolution with an isolated triplex system. Quantification of the temperature-dependent exchange of imino hydrogen atoms with solvent of the target DNA duplex allows determination of the changes in the stability of individual DNA duplex base pairs upon triplex formation. The data shown here investigates an antiparallel triplex, formed between the lncRNA hypoxia-inducible factor 1-α antisense RNA 1 (*HIF1α-AS1*) and the DNA target adrenomedullin (*ADM*), important in cardiovascular diseases. Triplex formation alters DNA structure and stability by affecting both hydrogen bonding strength and nucleobase-stacking interactions. These thermodynamic insights support bioinformatic methods to predict triplex stability and enhance our understanding of RNA•DNA:DNA triplex formation.

## INTRODUCTION

Long noncoding RNAs (lncRNAs) are nonprotein coding RNAs longer than 200 nt (Ponting et al. 2009). They play a crucial role in regulating gene expression and are often expressed in a tissue-specific manner (Filardi et al. 2020; Statello et al. 2021; Oo et al. 2022). LncRNAs have multiple modes of action, including the ability to interact with genomic DNA either by forming RNA:DNA heteroduplexes or RNA•DNA:DNA triplexes (Wang and Chang 2011; Kung et al. 2013; Kuo et al. 2019). RNA•DNA:DNA triplex formation has been reported for a variety of lncRNAs, including the maternally expressed gene 3 (*MEG3*), which regulates the TGF-β signaling pathway (Mondal et al. 2015). The *trans*-acting lncRNA hypoxia-inducible factor 1-α antisense RNA 1 (*HIF1α-AS1*), which is associated with various cardiovascular diseases, forms RNA•DNA:DNA triplexes with *Ephrin receptor A2* (*EPHA2*) and *Adrenomedullin* (*ADM*) genes that regulate key angiogenic functions (Leisegang et al. 2022; Warwick et al. 2023).

RNA•DNA:DNA triplexes are formed when RNA binds to the major groove of double-stranded DNA (dsDNA) through Hoogsteen or reverse Hoogsteen base-pairing. Two possible orientations are observed for the two different Hoogsteen interactions. Thus, RNA binding to the dsDNA can result either in a parallel orientation of the interacting DNA strand with pyrimidine-rich RNA and an antiparallel orientation with purine-rich RNA. While antiparallel triplexes are stabilized under neutral pH and in the presence of multivalent cations (e.g., Mg^2+^), parallel triplexes, if cytosines are involved, preferentially form under acidic conditions due to cytosine protonation, which facilitates Hoogsteen binding (de los Santos et al. 1989; Radhakrishnan et al. 1991; Duca et al. 2008; Bacolla et al. 2015; Li et al. 2016). In addition to the nucleobase composition, the length of the RNA strand also influences the stability of the triplexes. Kunkler et al. showed by in vitro stability studies that a length of at least 19 nt is necessary to form a stable RNA•DNA:DNA triplex, but increasing the length further did not markedly affect triplex stability (Kunkler et al. 2019). Recent in vitro biochemical and biophysical studies, including nuclear magnetic resonance (NMR) spectroscopy, have confirmed the formation of triplex structures (Ali et al. 2023; Warwick et al. 2023; Krause et al. 2024). It has been reported that triplex formation changes the structure of the B-form DNA duplex by altering the helical parameters, including the helical twist and rise, as evidenced by X-ray crystallography and solution NMR (Radhakrishnan and Patell 1993; Holland and Hoffman 1996; Gotfredsen et al. 1998; Rhee et al. 1999; Ruszkowska et al. 2020).

**FIGURE 1.**
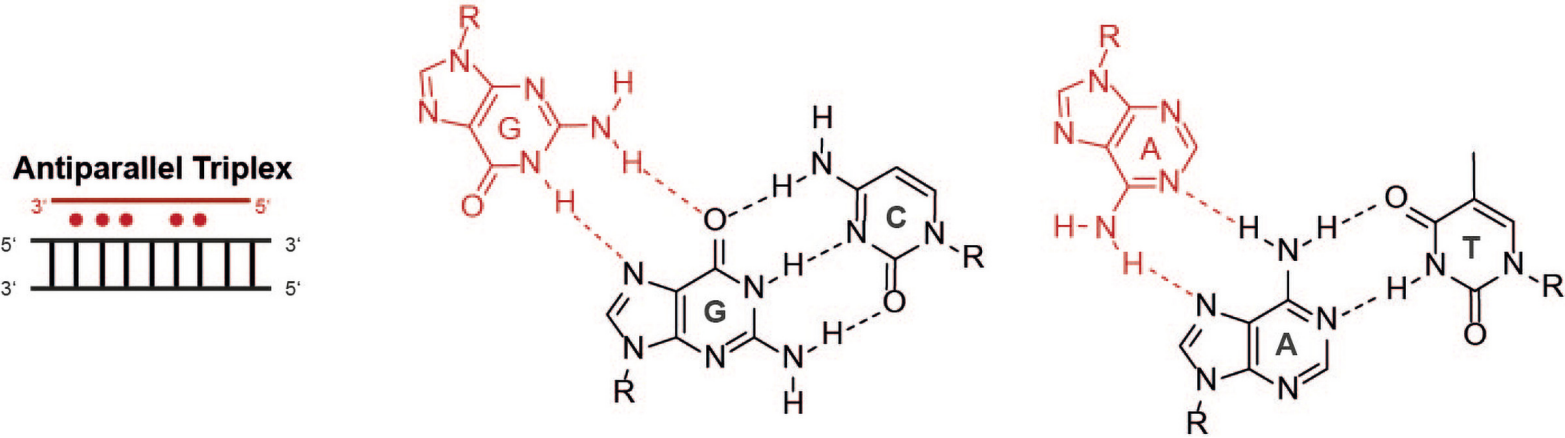
Base triplets that stabilize antiparallel RNA•DNA:DNA triplexes. (*Left*) Dots (•) indicate Hoogsteen interactions, lines (|) indicate Watson–Crick base interactions. The RNA binds to the purine-rich strand of the target DNA. (*Right*) DNA duplex nucleobases forming Watson–Crick base pairs are indicated in black, RNA nucleobases forming Hoogsteen interactions are indicated in red.

Stability and specificity of the major groove interaction of the lncRNA with the target duplex depend on Hoogsteen base-pairing stabilized by two hydrogen bonds (Fig. 1), which affects the Watson–Crick duplex hydrogen bonds. We previously examined triplex formation involving the triplex-forming oligonucleotide (TFO) of the lncRNA from *HIF1α-AS1* with a triplex DNA target site (TTS) from the *ADM* gene and observed changes in Watson–Crick base-pairing in the DNA duplex, induced by the Hoogsteen interaction with the RNA strand (Leisegang et al. 2022), in a qualitative manner. Triplex stability is concentration-dependent and matches the physiological temperature range.

The objective of this study is to determine the impact of triplex formation on the thermodynamics of individual Watson–Crick base pairs in the DNA target. We quantify the differences between temperature-dependent imino hydrogen (proton) solvent exchange rates of the DNA nucleobases in the duplex versus triplex and derived free energies (Δ*G*_diss_), enthalpies (Δ*H*_diss_), and entropies (Δ*S*_diss_) for individual base pairs, comparable with previous measurements on double-stranded DNA and RNA (Rinnenthal et al. 2010; Steinert et al. 2012; Wagner et al. 2015) and an intramolecular parallel triplex DNA (Powell et al. 2001). Interestingly, individual Hoogsteen base pairs act as stability hot spots. Although the thermodynamics of the weak Hoogsteen interactions cannot be directly observed, their effects on the target DNA can be investigated. The data obtained here provides a first quantitative information on the individual DNA base pairs. This quantitative information can in future augment further development of bioinformatics tools, such as *TriplexAligner* (Warwick et al. 2022), *Triplexator* (Buske et al. 2012) or *Triplex Domain Finder* (Kuo et al. 2019), to predict triplex stability that relies on sequencing data.

## RESULTS

### Imino proton exchange rates reveal base pair stability in the *ADM* DNA duplex and the triplex formed with *HIF1α-AS1*

**FIGURE 2.**
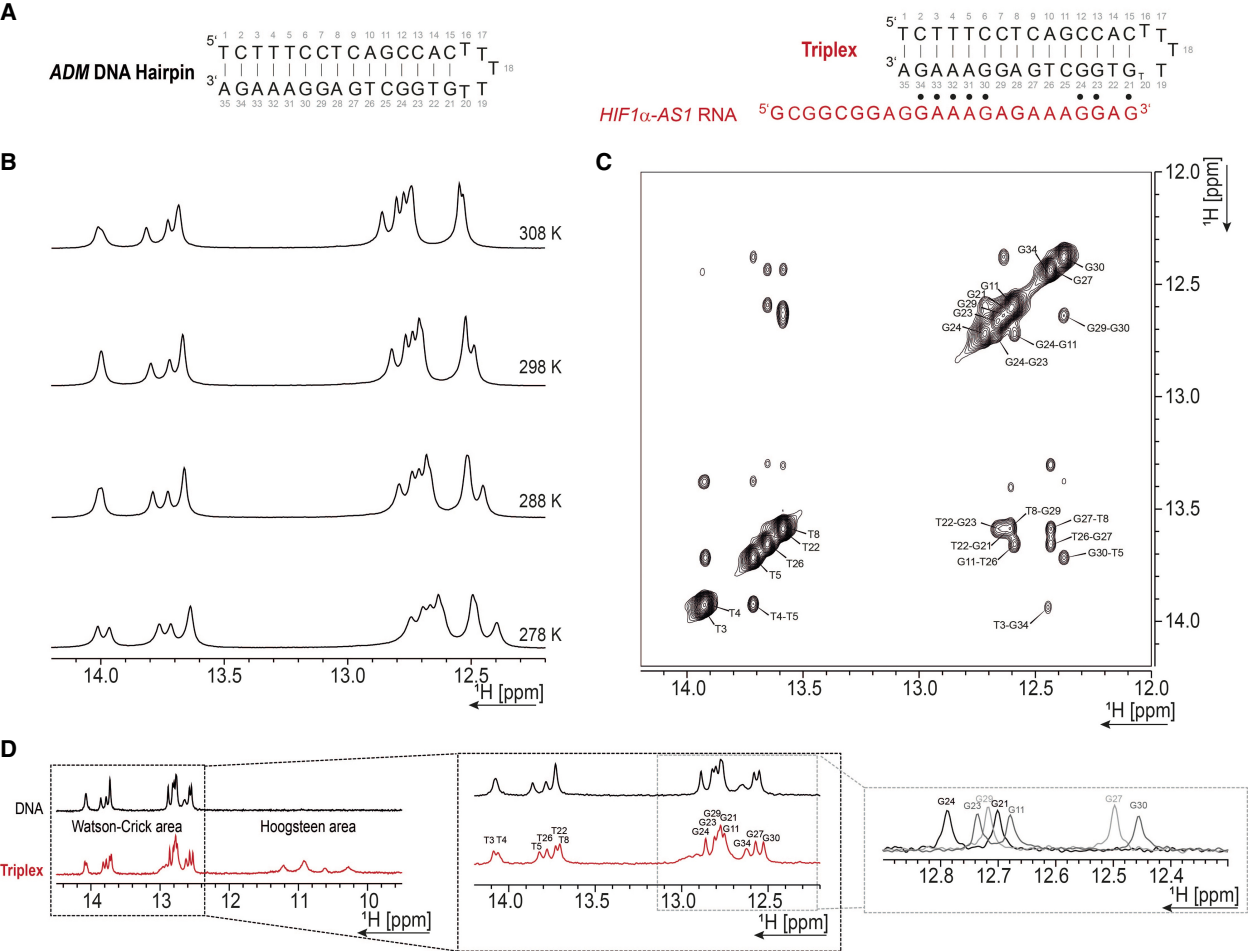
(*A*) Secondary structure of the *ADM* DNA hairpin in agreement with NMR measurements and the predicted secondary structure of the RNA•DNA:DNA formed between *HIF1α-AS1* RNA (red) and *ADM* DNA hairpin (black). (*B*) 1D ^1^H-NMR spectra temperature row of the *ADM* DNA hairpin (250 µM), measured from 278 to 308 K at 900 MHz. (*C*) [^1^H,^1^H]-NOESY spectra imino region of *ADM* DNA hairpin (250 µM). Sequential assignment is indicated. The sample was measured at 800 MHz, 200 msec mixing time at 288 K. (*D*) 1D ^1^H-NMR spectra of the *ADM* DNA only (black) and the triplex (red) imino region of the Hoogsteen base paired region and the Watson–Crick base paired region (*left*), enlargement of the Watson–Crick base paired region and the chemical shift assignment of the DNA bases (*middle*), and enlargement of the guanosine base region of the DNA hairpin measured with selectively labeled DNA samples to distinguish overlapping signals (*right*). NMR measurements were performed at 298 K. The NMR samples were measured in a buffer containing 25 mM HEPES-d_18_ (pH 7.4), 50 mM NaCl, 1 mM MgCl_2_ with 5% D_2_O with a sample concentration of 100 µM DNA and 300 µM RNA.

**FIGURE 3.**
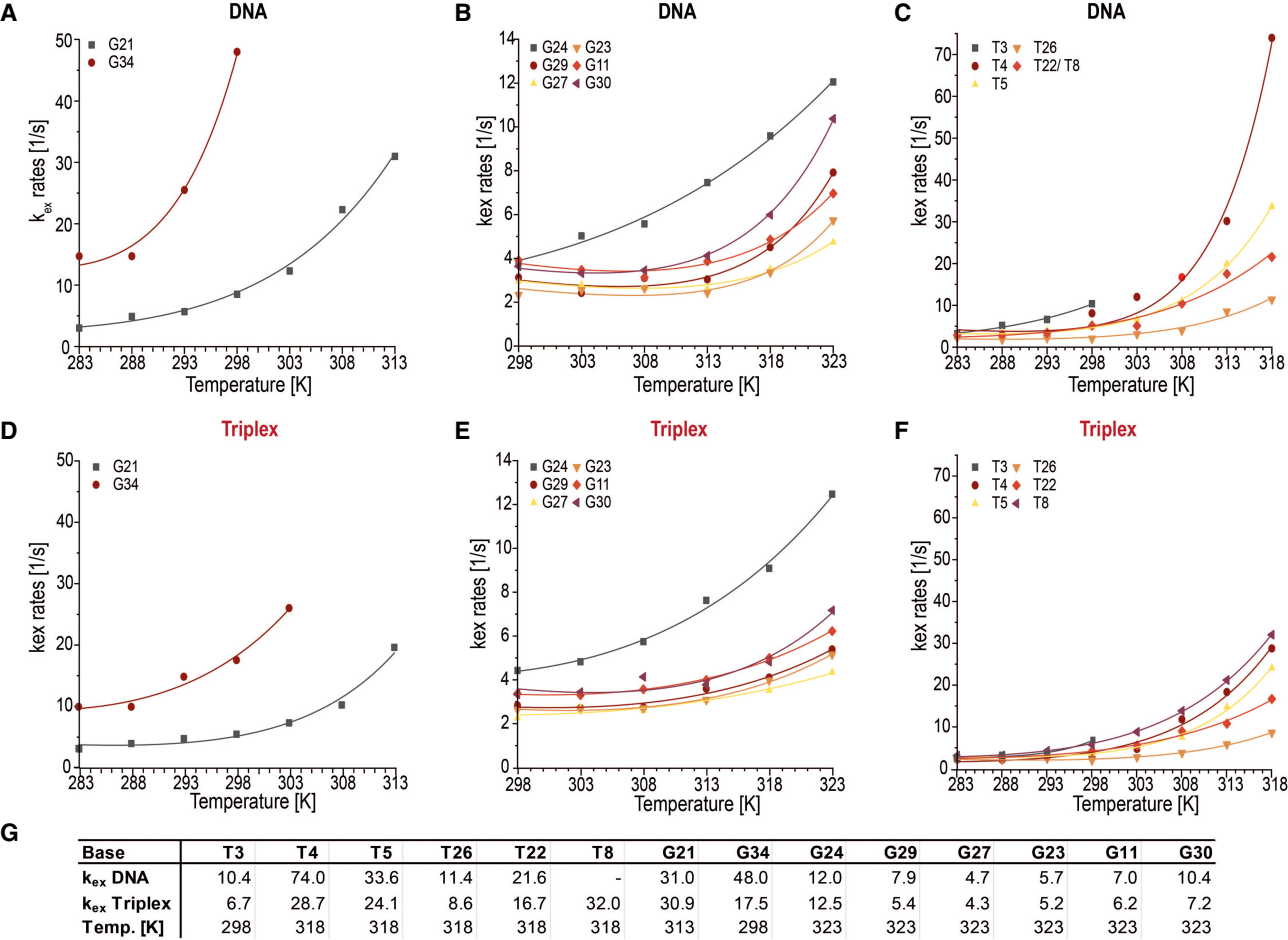
Exchange rates of the imino-hydrogen atoms with solvent water plotted against the temperature and fits for the terminal guanosines G34 and G21, the thymidines, and the guanosines in DNA (*A*–*C*) and the triplex (*D*–*F*). Overview of the highest *k*_ex_ values of the individual bases within the DNA and Triplex and the corresponding temperatures (*G*). NMR measurements were performed at 283–323 K, at 600 MHz. Samples were measured in a buffer containing 25 mM HEPES (pH 7.4), 50 mM NaCl, 1 mM MgCl_2_, and 5% D_2_O, with a sample concentration of 100 µM DNA and 300 µM RNA.

To compare individual DNA-DNA base pair stabilities of the *ADM* DNA target duplex (Fig. 2A, black) to those in the triplex formed with the TFO of the lncRNA *HIF1α-AS1* (Fig. 2A, red), imino proton solvent exchange rates *k*_ex_ were measured (see Materials and Methods). We used a DNA hairpin construct of the *ADM* DNA target so that the DNA–RNA interaction becomes bimolecular. Samples were prepared containing either only the DNA target or additional 3 eq of RNA to form the triplex (see Materials and Methods). Using ^1^H-1D (Fig. 2B) and 2D-^1^H, ^1^H NOESY NMR spectra (Fig. 2C), we sequentially assigned the imino protons of the DNA hairpin as a starting point for the determination of the individual base pair stabilities. In ^1^H-1D spectra, only peaks of thymidine imino protons, but not of deoxyguanosines, were well resolved. To overcome signal overlap in the spectral region of the guanosine imino proton signals, sequence-specific ^15^n labeled DNA samples were purchased from Innotope (Austria) (Fig. 2D, right; Table 2), to resolve exchange rates of individual deoxyguanosines imino sites. By inversion recovery NMR experiments, *k*_ex_ rates were determined and correlated with temperature (Fig. 3), revealing an overall difference between T-A and G-C base pairs, with significantly larger *k*_ex_ rates for T nucleobases in line with the reduced stability of T-A versus G-C base pairs (Fig. 3A,B vs. C, and D,E vs. F,G). G34 and G21 exhibited notably higher *k*_ex_ values compared to the other Gs (Fig. 3A vs. B, D vs. E,G) due to their sequence position. Notably, the overall *k*_ex_ rates are elevated in the DNA hairpin in comparison to the triplex sample.

### Thermodynamic stability of the base pairs reveals the impact of triplex formation on the DNA target

**FIGURE 4.**
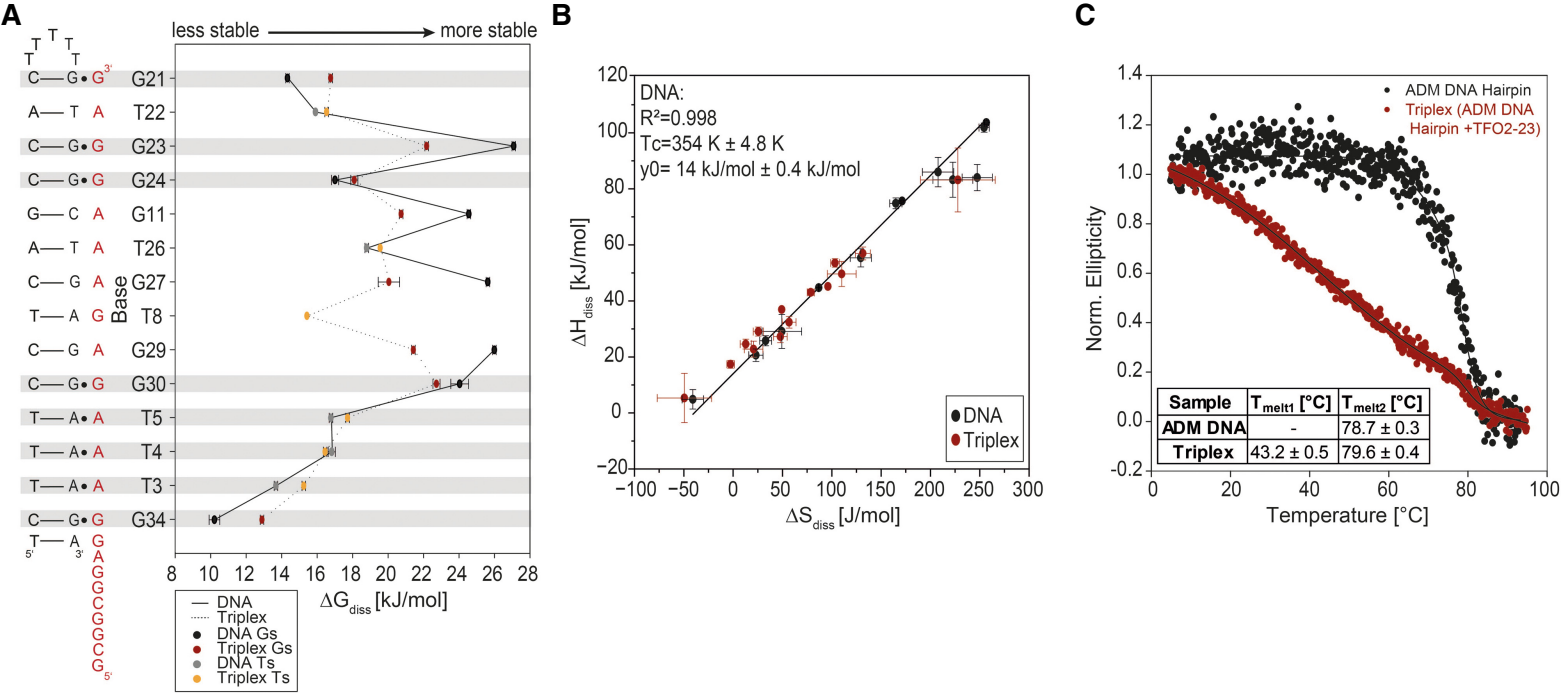
(*A*) Thermodynamic values of Δ*G*_diss_ for the Watson–Crick base pair opening in the DNA hairpin *ADM* and the triplex formed with *HIF1α-AS1* RNA. Predicted Hoogsteen base paired regions are highlighted in light gray. Values were determined by fitting *k*_ex_ against the temperature. Δ*G*_diss_ was calculated at 298 K. Error bars are indicated in black. (*B*) Δ*H*_diss_ (Δ*S*_diss_) correlation of the individual nucleobases in the DNA (black) and triplex (red). The compensation temperature (*T*_c_) and the intercept of the fit with the *y*-axis are indicated in the figure. (*C*) CD thermal melting analysis and determined melting points of the DNA hairpin (black) and the triplex (red). Sample was measured in a buffer containing 25 mM HEPES (pH 7.4), 50 mM NaCl, 1 mM MgCl_2_, with a concentration of 8 µM DNA hairpin, and additional 3 eq of RNA in the triplex sample.

To determine the enthalpic and entropic contributions to base pair stabilities, the dissociation enthalpies (Δ*H*_diss_) and entropies (Δ*S*_diss_) were determined by fitting k_ex_ versus temperature (Table 1), and the free enthalpies Δ*G*_diss_ were calculated using the Gibbs–Helmholtz equation (*T* = 298 K) (Rinnenthal et al. 2010). In general, higher dissociation free enthalpies Δ*G*_diss_ are a direct measure for high base pair stability. Inspection of the Δ*G*_diss_ values reveals that the values for T nucleobases are overall lower than those for G nucleobases (Fig. 4A; Triplex: orange vs. red dots, DNA: gray vs. black dots). The Δ*G*_diss_ values ranged between 10.3 and 27.1 kJ/mol. In direct comparison of the DNA hairpin and the triplex, a stabilization of the terminal bases G34, T3, T22, and G21 could be observed upon triplex formation. The Δ*G*_diss_ values of these bases were increased in the triplex sample (Fig. 4A). The potential causes of this effect include increased stacking interactions, which can reduce the fraying of DNA base pairs and promote cooperative helix stabilization.

**TABLE 1.** Thermodynamic values Δ*G*_diss,_ Δ*H*_diss_, and Δ*S*_diss_ for the Watson–Crick base pair opening in the DNA hairpin *ADM* and the triplex formed with *HIF1α-AS1* RNA

Base	Δ*H*_diss_ (kJ/mol) DNA	Δ*H*_diss_ (kJ/mol) triplex	Δ*S*_diss_ (J/mol) DNA	Δ*S*_diss_ (J/mol) triplex	Δ*G*_diss_ (kJ/mol) DNA	Δ*G*_diss_ (kJ/mol) triplex
G21	29.1 ± 6.1	49.6 ± 4.5	49.4 ± 20.0	110.1 ± 14.6	14.4 ± 0.1	16.8 ± 0.1
T22	25.8 ± 1.8	22.8 ± 2.8	33.0 ± 6.0	20.8 ± 9.4	15.9 ± 0.0	16.6 ± 0.1
G23	103.6 ± 0.6	36.9 ± 0.4	256.7 ± 1.8	49.3 ± 1.2	27.1 ± 0.1	22.2 ± 0.1
G24	4.8 ± 3.5	17.4 ± 1.4	−40.9 ± 10.8	−2.5 ± 3.9	17.0 ± 0.2	18.1 ± 0.2
G11	75.6 ± 0.3	24.6 ± 1.7	171.3 ± 1.2	12.8 ± 5.6	24.6 ± 0.1	20.8 ± 0.1
T26	44.7 ± 0.1	43.1 ± 1.1	86.8 ± 0.4	78.7 ± 3.7	18.8 ± 0.1	19.6 ± 0.0
G27	74.8 ± 1.9	5.3 ± 8.8	165.0 ± 6.2	−49.4 ± 27.5	25.6 ± 0.1	20.1 ± 0.6
T8	n.d.	32.4 ± 2.1	n.d.	56.7 ± 7.0	n.d.	15.5 ± 0.0
G29	101.9 ± 1.7	29.1 ± 1.6	254.5 ± 5.2	25.6 ± 5.1	26.0 ± 0.1	21.4 ± 0.1
G30	86.0 ± 5.2	53.6 ± 1.5	207.7 ± 15.9	103.4 ± 4.6	24.1 ± 0.5	22.7 ± 0.2
T5	55.4 ± 3.2	57.0 ± 2.3	129.4 ± 10.7	131.5 ± 7.6	16.8 ± 0.1	17.7 ± 0.1
T4	83.2 ± 6.2	45.1 ± 0.1	222.7 ± 20.2	96.1 ± 0.1	16.9 ± 0.2	16.5 ± 0.1
T3^a^	20.6 ± 2.2	83.2 ± 11.4	23.1 ± 7.3	227.8 ± 38.0	13.7 ± 0.1	15.3 ± 0.1
G34	84.0 ± 4.7	27.2 ± 2.1	247.4 ± 15.3	47.9 ± 7.0	10.3 ± 0.3	12.9 ± 0.1

Values were determined by fitting *k*_ex_ against the temperature. Δ*G*_diss_ were calculated using the Gibbs–Helmholtz equation with *T* = 298 K. (n.d.) Not determinable due to signal overlap in the NMR spectra.

^a^Signal detection only possible up to 298 K due to signal overlap in the NMR spectra.

It was found that base pairs directly involved in Hoogsteen base-pairing due to triplex formation were only slightly affected (Fig. 4A, highlighted in gray). Interestingly, the largest effects could be found for nucleobases that are not predicted to form Hoogsteen base pairs. Here, the DNA hairpin showed overall higher Δ*G*_diss_ values for G11, G27, and G29 compared to the triplex (Fig. 4A). Even in regions where RNA does not form Hoogsteen interactions, adjacent triplex formation can destabilize Watson–Crick base pairs. This is believed to be due to changes in the helical properties of the DNA, altered base stacking, and entropic constraints imposed by the global triplex architecture. This demonstrates that RNA binding exerts indirect effects on DNA stability beyond direct contact.

### Enthalpy-entropy correlation of base pair dissociation reflecting DNA hairpin melting transitions

The enthalpic and entropic parameters (Table 1) associated with base pair dissociation show a linear correlation. Similar enthalpy-entropy correlations have been previously reported for nucleic acid base pair dissociation (Rinnenthal et al. 2010; Steinert et al. 2012) and interpreted as solvent-induced hydration property (Lumry and Rajender 1970; Strazewski 2002; Rinnenthal et al. 2010). Figure 4B shows the relationship between Δ*H*_diss_ and Δ*S*_diss_ for both the DNA hairpin and the triplex structure. The slope of this correlation leads to the compensation temperature (*T*_c_) and is commonly interpreted as arising from solute-solvent interactions. The intercept with the *y*-axis, however, is interpreted to result from solute-intrinsic factors, primarily the average base-stacking free energy per nucleobase (Rinnenthal et al. 2010; Steinert et al. 2012). The intercept with the *y*-axis revealed a *y*_0_ value of 14 kJ/mol for the DNA hairpin.

The Δ*H*_diss_(Δ*S*_diss_) correlation of the DNA hairpin resulted in *T*_c_ = 354 K ± 4.8 K, which is remarkably close to the melting temperature determined from the thermal melting assays (Fig. 4B,C) showing a thermal melting point *T*_melt_ = 78.7°C ± 0.3°C (351.7 K) of the DNA hairpin. These melting temperatures are in the range of those predicted using the DINAMelt web server (Markham and Zuker 2005), where a melting temperature between 355.15 and 358.35 K was calculated.

For the triplex sample, a *T*_melt_ = 79.6°C ± 0.4°C (∼353 K) for the DNA hairpin melting and a broader melting transition around 43°C (∼316 K) could be detected by CD melting, corresponding to the stepwise dissociation of single-stranded RNA (Fig. 4C) from the triplex.

## DISCUSSION

Our experiments show the effect of triplex formation detected on the DNA target site of *ADM*. Surprisingly, the expected weakening of DNA target Watson–Crick base pairs was more pronounced in regions not directly engaged in Hoogsteen interactions with the TFO, than in the regions that are directly involved in Hoogsteen base-pairing. For those sites, triplex formation led to destabilization of G-C base pairs compared to the DNA hairpin, supporting previous reports that triplex formation can affect the geometry and change helical properties compared to B-form DNA (Rhee et al. 1999; Ruszkowska et al. 2020). Thus, the effect on the stability is not restricted to Hoogsteen H-bond formation, but stacking interactions are important stabilizing forces in triplex formation, previously not taken into account. In agreement with this, the aromatic base resonances (Supplemental Fig. S1) show detectable chemical shifts upon triplex formation, indicating changes in the local environment caused by base stacking. The enthalpy-entropy correlation of base pair opening in the DNA hairpin yielded a compensation temperature (*T*_c_ = 354 K ± 4.8 K) matching the melting transition determined by thermal melting assay, and with an ordinate intercept corresponding to an average base-stacking free energy of ∼14 kJ/mol.

We could here establish the alteration of individual DNA target base pairs upon interaction with the TFO *HIF1α-AS1*. Further studies aiming to expand such studies to the Hoogsteen side interaction, is hindered by only weak NMR signals of the involved imino hydrogens (Fig. 2D). The Hoogsteen interactions are too weak. Thus, more detailed NMR structure determination cannot be conducted in solution. We thus use the indirect DNA duplex reported signals to characterize Hoogsteen interaction on DNA base pairs.

We conducted experiments on a single triplex system. Expanding the approach to additional systems is certainly desirable. For such future studies, we wish to delineate the time requirements for the indicated experiments. The temperature dependence of the imino exchange was characterized for nine temperatures ranging from 283 to 323 K. The measurement time required for this type of analysis is ∼21 h for a single sample. However, this approach did not provide sufficient signal resolution for the G nucleotides. To address the challenge of signal overlap, the investigation of samples with specific deoxyguanosine isotope labeling schemes was essential. Three ^15^N1 isotope-labeled DNAs were purchased at a total cost of approximately €2000 for each sample. The measurements were optimized with x-filtered experiments to separate signal of imino hydrogen either bound to ^14^n or ^15^n. Each sample required a total measurement time of 36 h. Six samples were measured over a period of 216 h. The samples included three replicates of DNA with different labeling schemes and three replicates of DNA plus RNA, resulting in triplex formation.

In this article, we present a comprehensive procedure for measuring the stability of individual base pairs and the resulting thermodynamics for triplexes within an isolated system. These results demonstrate that, in the isolated triplex system investigated in this study, antiparallel triplex formation alters DNA structure and stability, providing fundamental insight into how such structures can influence their DNA target sites. The data reported here provide an initial reference point for improving prediction tools for triplex stability and for understanding the thermodynamic factors that stabilize RNA•DNA:DNA triplexes, thereby enhancing specificity and selectivity. While these results are based on a single system, they establish a foundation upon which future studies incorporating additional data sets can build toward a more comprehensive and broadly applicable understanding.

## MATERIALS AND METHODS

### Sample preparation

Unlabeled RNA and DNA constructs were purchased by Horizon and purified following the manufacturer's instructions. The samples were purified by in-house HPLC. Following HPLC purification, the DNA samples were precipitated with isopropanol and the RNA samples with LiOCl_4_. Isotope labeled RNA (Table 2) for further NMR experiments with filtered pulse sequences (see below) were purchased by Innotope (Austria) and purified following the manufacturer's instructions. The DNA and RNA samples were then desalted with centrifugal concentrators (Sartorius AG).

**TABLE 2.** Sequences of the DNA and RNA construct used for NMR experiments

Construct name	Sequence (5′-3′)
*ADM*_CTGA unlabeled	TCTTTCCTCAGCCAC TTTTT GTGGCTGAGGAAAGA
*ADM*_CTGA ^15^N G21; G24	TCTTTCCTCAGCCAC TTTTT GTGGCTGAGGAAAGA
*ADM*_CTGA ^15^N G27; G29	TCTTTCCTCAGCCAC TTTTT GTGGCTGAGGAAAGA
*ADM*_CTGA ^15^N G11; G23; G30	TCTTTCCTCAGCCAC TTTTT GTGGCTGAGGAAAGA
*HIF1α-AS1* RNA	GCGGCGGAGGAAAGAGAAAGGAG

^15^N1-labeled bases are indicated in red.

Triplex samples were hybridized following the protocol of Kalwa et al. (2016) and as described in the following: RNA•DNA:DNA triplexes were formed by the addition of 3 eq of RNA to the DNA hairpin, incubated at 60°C for 1 h, and then allowed to cool slowly to room temperature. DNA hairpins were used to form more stable triplex samples, as reported previously (Leisegang et al. 2022; Krause et al. 2024). Samples were hybridized in a triplex buffer containing 25 mM HEPES-d_18_, 50 mM NaCl, and 1 mM MgCl_2_ (pH 7.4).

### NMR spectroscopy

To characterize individual base pair stabilities of the duplex DNA in comparison to the triplex, two types of samples were prepared. One sample with only DNA hairpin, another one with the RNA•DNA:DNA triplex for all the three DNA sequences (Table 2). Both sample types were prepared in an aqueous triplex buffer containing 5% D_2_O. For this approach, we measured ^1^H-detected inversion recovery experiments with varying exchange delays (4 µsec–4 sec). NMR experiments were performed as described in Rinnenthal et al. (2010) NMR experiments were performed in the BMRZ (Center for Biomolecular Magnetic Resonance) using Bruker spectrometers with 600 MHz to determine the solvent imino exchange rates. Measurements were performed at 283–323 K.

### Solvent exchange rates and thermodynamic analysis

The intensities obtained were plotted against the delay and fitted with the equation from Rinnenthal et al. (2010). Before plotting and fitting, all intensities were normalized to the first delays absorbance (*I*(*x*)/*I*(4*u*)). Using Equation 1 allowed the determination of *k*_ex_ by fitting the imino proton signal intensities (*I*_n_) as a function of the mixing time *τ*_m_.

(1) $$\frac{I_{n}(\tau_{m})}{I_{n}(0)}-1=\left(\frac{I_{w}(0)}{I_{n}(0)}\right)\cdot k_{\mathrm{ex}}\cdot \frac{\exp(-R_{1n}\cdot t)-\exp(-R_{1w}\cdot t)}{-R_{1n}+R_{1w}}$$

*I*_*n*_(*τ*_m_) = intensity of the imino proton at time point *τ*_m_
*I*_*n*_(0) = intensity of the imino proton at time point *τ*_m_ = 0
*R*_1*w*_ = *R*_1_ relaxation rate of the water
*R*_1*n*_ = *R*_1_ relaxation rate of the imino protons
*k*_ex_ = exchange rate between imino protons and bulk water
*R*_1*w*_, *R*_1*n*_, and *k*_ex_ were allowed to adjust freely.

The *k*_ex_ values received from the fit were plotted against the temperature to estimate the thermodynamic parameters, enthalpy Δ*H* and entropy Δ*S*, for calculating the Gibbs Energy Δ*G* with Equation 2 (Rinnenthal et al. 2010).

(2) $$k_{\mathrm{ex}}(T)=\frac{\left(\frac{k_{B}T}{h}\right)\cdot \frac{\exp(-(\Delta H_{TR}-T\cdot \Delta S_{TR}))}{RT}}{\left(1+\frac{\exp(\Delta H_{\mathrm{diss}}-T\cdot \Delta S_{\mathrm{diss}})}{RT}\right)}+d(T)$$

with Δ*H*_diss_ − *T* · Δ*S*_diss_ = Δ*G*_diss_
*k*_ex_(*T*): exchange rate at temperature T
*k*_*B*_: Boltzmann constant (1.38·10–23 J/K)
*h*: Planck's constant (6.63·10–34 J)
*R*: gas constant [8.31 J/(mol K)]
Δ*H*_tr_: transition state enthalpy
Δ*S*_tr_: transition state entropy
*d*(*T*) accounts for cross-polarization effects.

Errors for Δ*H*_diss_ and Δ*S*_diss_ were obtained from the fit. Errors for Δ*G*_diss_ were calculated as the mean of the boundary values, with the error reported as the standard deviation.

## DATA DEPOSTION

Experimental raw data have been deposited under https://doi.org/10.25716/gude.0kxc-43mg.

## SUPPLEMENTAL MATERIAL

Supplemental material is available for this article.
